# Individual and environmental factors associated with cognitive function in older people: a longitudinal multilevel analysis

**DOI:** 10.1186/s12877-022-02940-9

**Published:** 2022-03-23

**Authors:** Hui-Chuan Hsu, Chyi-Huey Bai

**Affiliations:** 1 School of Public Health, Taipei Medical University, Taipei, Taiwan (R.O.C.); 2 Research Center of Health Equity, College of Public Health, Taipei Medical University, Taipei, Taiwan (R.O.C.); 3 Department of Public Health, College of Medicine, Taipei Medical University, Taipei, Taiwan (R.O.C.)

**Keywords:** Cognitive function, Multilevel analysis, Longitudinal study, Social support, Older people

## Abstract

**Background:**

Individual and environmental factors have been found to be related to cognitive function. However, few studies have examined the longitudinal effects of both individual and environmental factors over time. The purpose of this study was to examine the effects of individual and environmental factors over time on older people’s cognitive function.

**Methods:**

Nationally representative panel data from the Taiwan Longitudinal Survey on Aging 1999–2015 (*n* = 6349 persons, observations = 12,042) were used. City-level indicator data were sourced from the government. A multilevel mixed linear model analysis was conducted.

**Results:**

Better cognitive function was significantly related to individuals’ work, ethnicity, younger age, higher education level, better self-rated health, higher level of emotional support received, being more religious, higher economic satisfaction, and living in the cities with higher population densities. Education and social connectedness were protective factors over time.

**Conclusion:**

Socioeconomics and social connectedness are related to cognitive function. A more social integrated lifestyle and financially secure living is suggested in the policy.

**Supplementary Information:**

The online version contains supplementary material available at 10.1186/s12877-022-02940-9.

## Introduction

The number of people living with dementia worldwide is over 50 million and is expected to rise to 152 million by 2050 [1]; cognitive impairment impacts the quality of life of older adults and their families. Existing cross-sectional and longitudinal studies have found related individual factors related to cognitive impairments, including genetic, disease-related, lifestyle-related, demographic, and social factors. Cognitive function may also decline over time due to interactions between older individuals’ risk factors. In recent studies, environmental factors, such as air pollution, social and built environments, and population composition in living areas, have also been found to be related to cognitive function [2]. Although the influence of individual and environmental factors on cognitive function has both been explored and longitudinal data for individuals have been collected, there is little research that takes into account person, place, and time effects together. Contextual factors may also change with time and interact with an individual’s time-varying factors during the life course. In this study, we used longitudinal nation-representative individual data of older adults linked to city-level indicators across time to examine the individual-, time-, and area-level factors and their interactions on cognitive function in older adults.

According to the ecosystem theory [3], different level factors (microsystem, mesosystem, exosystem, and macrosystem) may affect an individual’s health, including cognitive function. Microsystem factors are individual factors, such as sex, socioeconomic status [2, 4–8] and education [4–6], genetic factors [9–14], nutrition status [15, 16], metabolic syndrome [17], physical function difficulties [7, 8, 18], frailty and sarcopenia [19, 20], physical activity and exercise [21, 22], social activity [23], retirement [24] and religiousness [25]. Genetic factors may also interact with behavioral factors and affect cognitive function. The effect of physical exercise on cognitive performance in older adults is also conditioned or moderated by the presence of apolipoprotein E (ApoE4) [12, 13], a genetic risk factor in cognitive impairment. Dietary habits such as protein or fiber intake showed a similar pattern [14]. Mesosystem factors, i.e., interpersonal relationships, related to cognitive function include social networks and social relationships [26–28] and stress [29]. Exosystem factors indicate the living environment. Living environment factors related to cognitive function included nature and physical environment (such as air and industry pollution) [4], social abnormalities (such as safety and cleanliness) [6], perceived safety and social cohesion [28], population composition [6, 7], socioeconomic status of the community, built environment and social characteristics [2, 7, 8, 30, 31]. Macrosystem represents policy and social context. Population education and gross domestic product (GDP) are positively related to cognitive function [32]. Usually the microsystem factors and the mesosystem factors were categorized as individual factors, while exosytem factors and macrosystem factors were viewed as the environmental factors.

Environmental factors can also be categorized compositional or contextual [33]. Compositional effects indicate the heterogeneity of areas, and such population age structure or educational compositions [6, 7]. Contextual effects refer to the social and physical environment with which individuals interact and accordingly affect health [4, 6, 28].

A longitudinal study design with repeated measures is suggested to examine the interaction of time effects with related factors because cognitive function normally declines over time with aging. Existing research has explored the interaction of time effects with covariates and its effects on cognitive function. Lower socioeconomic status was related to declining cognitive function over time [5], while having a spouse, exercising, having a larger social network, and driving as the means of transportation may be protective factors for cognitive function over time [5]. Zaninotto et al. [18] found that women declined faster than men in memory, executive function, and global cognitive function, and increasing age and dementia predicted a faster decline. Brown et al. [23] used four longitudinal data sets and found that social activity was more related to fluency and memory over time, but not all the domains of cognitive function. A longitudinal study from China indicated that older people who lived in urban communities had higher cognitive function than those who lived in rural communities, but the cognitive function of those who lived in urban communities also declined faster [34].

Person and environmental factors may also affect cognitive function. People living in medium–high population areas had better cognition, but those with disabilities living in the densest population areas had worse cognitive function [35]. Community infrastructure has a greater impact on cognitive function in rural areas than in urban areas [8].

Although area-level characteristics are found to be related to cognitive function, and many longitudinal studies using individual data have been conducted, there are only a few longitudinal studies analyzing area-level characteristics and individual factors changing over time, and the results are not consistent. Clarke et al. [5] examined the trajectory of cognitive function and relationship with neighborhood characteristics over 18 years. The neighborhood characteristics (such as community center, public transition, and public space) were not significant at the intercept, but these neighborhood factors were significantly related to declining cognitive function over time. Residence surrounding green space was related to better cognitive function over time [36] because green lands may foster physical activity and social interaction. Luo et al. [8] found that the physical characteristics (such as accessibility and bus lines) and the social characteristics (such as employment services and community socioeconomic status) of neighborhoods were protective of cognitive function over time. However, the interactions of individual factors with time and person with environment were not included. Meyer et al. [37] and Wörn et al. [38] found that higher neighborhood socioeconomic status was related to better cognitive function at the intercept (baseline), but neighborhood socioeconomic status was not significant on the time slope.

Older people’s cognitive function is related to individual characteristics, environmental factors, and time. However, the interactions of these three domains are not always simultaneously examined. In this study, we used longitudinal data in the case of Taiwan to explore the individual factors and environmental factors over time to examine the effects of persons and environment over time on cognitive function in older people.

## Methods

### Data and Sample

The data for this study combined longitudinal data of older adults and city-level indicators from the government. The longitudinal data were from the Taiwan Longitudinal Survey on Aging (TLSA), which is nationally representative of older people aged 60 years old or older since 1989 and collected every 3 or 4 years. The new cohorts of the supplement samples were added in the follow-ups. Proportional-to-size sampling was conducted, and the questionnaires were conducted by face-to-face interviews. The current study used the sample from 1999 to 2015, and only those who completed the cognitive function questions were included for analysis. In total, the analysis sample included 6349 persons with 12,042 observations (please see Supplementary Figure S1). The TLSA data were deidentified when released. City-level data were from the open data of the government. The study obtained approval from the Taipei Medical University Joint Institutional Review Board (N201912135) before the study was conducted.

### Measures

Area-level indicators were selected based on a literature review, and the source was open data from the government. Because most of the available open data were provided based on cities, the unit of the area was the city.

#### Measures: Individual variables

- 1. The cognitive function items across waves of TLSA were not consistent, and therefore only the common items were used. Eight items were from Short Portable Mental Status Questionnaire (SPMSQ) [39]; the other two items from the Montreal Cognitive Assessment (MoCA) [40] and verbal memory test [41]. The items were as following: where are you, what day is today, what’s the day of the week, how old are you, mother’s maiden name, current president, last president, 20 minus 3 consecutively, reverse a series of numbers, and recall 10 items. The total score ranged from 0 to 19.
- 2. Demographic variables included age, sex, current residential city, education (illiterate, no formal education or elementary school, primary high school, senior high school, college or university and above), marital status (having a spouse or not), ethnicity (Fuchien, Hakka, mainlanders, and aboriginal and others), having children (yes/no), living arrangement (living with others or alone), number of types of social contact at least once per week (parents, brothers and sisters, other relatives, friends and neighbors), and economic satisfaction (score 1-5).
- 3. Health behaviors inlcued smoking (current smoker, quitted smokers, nonsmoker) and drinking frequency (0-5).
- 4. Health conditions included self-rated health, chronic disease numbers, advanced physical function difficulties [42], disability number in activities of daily living (ADLs) [43], disability number in instrumental activities of daily living (IADLs) [44], depressive symptoms, and stress. Self-rated health was scored from 1 to 5, indicating very poor to excellent. The chronic disease number was defined as the current morbidity of the following diseases: hypertension, diabetes, heart disease, stroke in treatment, cancer, respiratory disease, arthritis, ulcer, liver or gallbladder disease, kidney disease, and gout. Advanced physical function difficulties were measured by the Nagi physical function scale (each item was scored 0-3, and the total score was 0-27) [42]. The disability number in ADLs included the items of eating, dressing, transferring, going to the toilet, taking a bath, and walking indoors. Each item with which the person had difficulty for at least 3 months was defined as a disability; the ADL disability number was the cumulative disability numbers of 6 items. The disability number in IADLs included the following items: shopping for groceries, managing money, going out by car/train alone, heavy housework, light housework, and making a phone call. The IADL disability number was defined as the cumulative difficulty of the items. Depressive symptoms were measured using the Center for Epidemiological Studies Depression 10-item scale (CESD-10) [45]; the total score ranged from 0 to 30. Stress was measured by stress or disturbance in the following items: self’s health; financial status; job; family member’s health, financial status, job, or marriage; family relationship; and others. Each item was scored from 0 to 2, indicating no stress, a little, or a great deal of stress; the total score was from 0 to 12.
- 5. Regarding social participation and social support, work was defined as yes/no. Social support included providing instrumental support (helping in ADLs or IADLs for family or taking care of children, scored 0-6); receiving instrumental support (family/friends they can rely on when sick, scored 1-5; available help, scored 0 or 1; total score was 0-6); and receiving emotional support (family/friends listen to you, care about you, their care is satisfactory; each item was scored from 1 to 5, and the total score was from 3-15). A social group was defined as participating in any of the following groups (yes/no): community social interaction groups, religious groups, association or business organization, political party, clan groups, older people’s clubs, older people’s college, and volunteer groups. Religiousness was measured by 4 items for those who had religion belief: pray or worship at home, reading sutras or the Bible, going to temple/church, and watching/listening to religion programs; each item was scored from 1 to 4 according to its frequency. Those who did not have religious beliefs were defined as 0. The total score was from 0-16.

### Measures: City-level indicators

City-level data were obtained from the open data of the government. Because some of the participants migrated to different cities across waves, the city-level data were defined as data from the city where participants lived during the survey year. The indicators were selected based on literature review [2, 6–8, 30, 31], included the following: (1) Population characteristics: population density (100 people per kilometer square), percentage of the population with a high level education (college or university and above) (%), percentage of the population who were older people (age 65 and above) percentage of the population (%); (2) Medical resources and built environment: medical personnel numbers (per 10 thousand population), hospital beds (per 10 thousand population), and green area for leisure purposes (hectares per 10,000 persons); and (3) Personal and financial security: crime rate (per 100 thousand population), percentage of the population considered low-income, median income (NT dollars), unemployment rate (%), and household income Gini coefficient (the household income of the city was categorized into 5 groups, and then the Gini coefficient was calculated; higher Gini coefficients indicated a more diverse income range among the households with the lowest and the highest income). In total 22 cities were included for analysis.

### Analysis

The analysis methods used in this study included descriptive analysis, bivariate analysis of independent variables and cognitive function, and multilevel linear mixed model analysis. The effects of the independent variable on the intercept and time slope were estimated for the repeated time-varying variables. Level 1 of the model represents the changes of the individuals or cities over time. Level 2 of the model represents the differences across individuals at the intercept level. Level 3 of the model represents the differences at the city level. Because the individual factors and the city factors were both time-varying, we used repeated measures of the data and added the individual factors, the city factors, time, the time-individual interactions, and the time-city interactions in the model as the fixed effects. Random effects were assumed on the repeated measures within individuals and intercepts at the cities. The analyses were conducted by SPSS Statistics 22 software (IBM, SPSS Inc., Chicago, IL, USA) by using the command GENLINMIXED (generalized linear mixed models [46].

## Results

Table 1 shows the descriptive analysis of the participants’ characteristics over the five waves (1999, 2003, 2007, 2011, and 2015). Although the participants were not the same, average cognitive function declined over time because of aging.

**Table 1:** Descriptive analysis of the sample across 5 waves of TLSA, 1999–2015

	1999 (*n* = 1624)	2003 (*n* = 2847)	2007 (*n* = 2480)	2011 (*n* = 2418)	2015 (*n* = 2673)
Cognitive function (0–19)	12.43 (2.93)	12.20 (2.83)	12.30 (2.28)	12.02 (2.45)	11.00 (3.29)
Sex: Male	47.8%	48.8%	49.6%	49.9%	48.9%
Female	52.2%	51.2%	50.4%	50.1%	51.1%
Marital status: Having spouse	78.4%	78.7%	80.4%	277%	90.3%
No spouse	21.6%	21.3%	19.6%	72.3%	9.7%
Children: yes	100.0%	96.7%	96.9%	96.7%	100.0%
no	0.0%	3.3%	3.1%	3.3%	0.0%
Living arrangement: With others	93.3%	4.6%	7.7%	7.8%	90.4%
Alone	6.7%	95.4%	92.3%	92.2%	9.6%
Smoking: no	68.3%	66.5%	65.7%	67.3%	68.8%
Smoking: quitted	10.6%	12.3%	15.7%	18.9%	19.4%
Smoking: current	21.1%	21.2%	18.6%	13.8%	11.9%
Exercise: Irregular	54.0%	51.4%	52.9%	46.9%	54.5%
Regular	46.0%	48.6%	47.1%	53.1%	45.5%
Work: yes	44.3%	48.6%	40.8%	36.0%	22.5%
No	55.7%	51.4%	59.2%	64.0%	77.5%
Social group participation: Yes	52.0%	45.0%	49.0%	49.1%	44.2%
No	48.0%	55.0%	51.0%	50.9%	55.8%
Ethnicity: Fuchien	68.4%	70.5%	70.5%	71.1%	71.3%
Ethnicity: Hakka	17.7%	18.4%	18.5%	18.3%	18.4%
Ethnicity: Mainland	12.5%	09.4%	9.6%	9.2%	8.6%
Ethnicity: others	1.4%	1.7%	1.4%	1.4%	1.6%
Age 50–54	9.2%	32.2%	7.9%	0.0%	0.0%
Age 55–59	28.0%	17.7%	31.0%	16.2%	0.0%
Age 60–64	22.8%	16.3%	16.6%	28.7%	22.0%
Age 65–69	14.3%	12.4%	16.2%	17.0%	25.0.%
Age 70–74	18.6%	9.3%	11.2%	14.2%	16.9%
Age 75–59	6.2%	9.2%	9.4%	10.0%	14.6%
Age 80–84	0.7%	2.6%	6.2%	9.2%	9.7%
Age 85 +	0.2%	0.4%	1.7%	4.5%	11.8%
Education: illiterate	26.5%	16.6%	13.5%	13.1%	15.1%
Education: elementary school	48.4%	48.5%	48.5%	48.4%	48.3%
Education: junior high school	9.8%	12.0%	12.9%	13.1%	12.3%
Education: Senior high school	8.4%	12.2%	13.5%	13.5%	13.0%
Education: college/univ. +	6.8%	10.6%	11.5%	11.9%	11.3%
Drinking frequency (ordinal)	0.77 (1.49)	0.28 (0.90)	0.25 (0.87)	0.24 (0.82)	0.27 (0.63)
Disease number	0.99 (1.18)	1.04 (1.19)	1.17 (1.26)	1.32 (1.26)	1.42 (1.31)
Self-rated health	3.43 (1.02)	3.43 (1.05)	3.32 (0.94)	3.27 (0.95)	3.21 (0.96)
ADL disability number	0.03 (0.31)	0.04 (0.37)	0.06 (0.48)	0.14 (0.75)	0.28 (1.06)
IADL disability number	0.31 (0.81)	0.32 (0.84)	0.40 (0.96)	0.36 (1.40)	0.74 (2.04)
Advanced function difficulties	2.08 (3.81)	2.26 (2.38)	2.38 (4.35)	3.14 (5.13)	4.35 (6.26)
Contact outside households	2.86 (1.08)	2.15 (1.09)	1.94 (0.96)	2.16 (0.97)	2.26 (1.06)
Depressive symptoms	4.19 (4.98)	4.05 (4.95)	3.96 (5.10)	4.04 (5.27)	4.62 (5.44)
Stress	1.71 (1.99)	1.84 (1.99)	4.35 (1.42)	2.10 (2.25)	1.93 (2.11)
Providing instrumental help	0.51 (0.89)	0.62 (1.03)	0.49 (0.88)	0.67 (1.31)	0.49 (1.16)
Receiving emotional support	12.44 (2.25)	12.42 (2.17)	12.51 (2.01)	12.42 (2.02)	12.40 (2.10)
Receiving instrumental support	5.16 (1.00)	5.16 (1.00)	5.12 (0.97)	5.20 (0.94)	5.19 (0.90)
Economic satisfaction	3.17 (0.91)	3.09 (0.99)	3.16 (0.93)	3.25 (0.93)	3.39 (0.83)
Religiousness	8.32 (3.94)	8.39 (3.82)	8.16 (3.99)	8.27 (3.86)	7.92 (3.73)

The descriptive analysis of the 22 city levels across 5 waves are shown in the Supplementary Table S1. Most of the city indicators increased over time, while the unemployment rate and green land area fluctuated across waves. Some of the city-level indicators were highly correlated (r > 0.7), such as population density and highly educated population, medical personnel and hospital beds, highly educated population and median income, unemployment rate and crime rate, and highly educated population and median income. Some of the city-level factors were highly correlated (please see Table S2). To avoid collinearity, high education population and medical personnel were not included in the linear mixed model analysis.

We assumed that the multilevel analysis included repeated measures across time, individuals, and cities, and the model was assumed to be a time-dependent linear model. The fixed effects included individual factors at the intercept, individual factors at the time slope, city factors at the intercept, and city factors at the time slope. The random effects of repeated measures and individuals were also included. The model of individual factors with time and the model of city factors with time were first analyzed to test the significance (please see Table S3), and then the significant variables at the intercept were included in the final model in Table 2.

**Table 2:** Multi-level mixed linear modeling of older adults’ cognitive function with individual and city indicators by TLSA 1999–2015

Variables	B (SE)	95% C.I	
**Fixed effects**			
**Individual-level**			
Intercept	9.799 (0.573)***	8.677	10.9216
Sex (male)	0.144 (0.104)	-0.059	0.347
Work (yes)	0.294 (0.097)**	0.103	0.485
Ethnicity (Hakka)	0.115 (0.119)	-0.118	0.348
Ethnicity (mainlander)	0.091 (0.168)	-0.238	0.420
Ethnicity (others)	-1.361 (0.332)***	-2.013	-0.709
Age (ordinal)	-0.205 90.032)***	-0.267	-0.143
Education (ordinal)	0.631 (0.045)***	0.543	0.720
Self-rated health	0.174 (0.044)***	0.087	0.260
IADL disability	-0.231 (0.052)***	-0.333	-0.128
Contact outside households	0.016 (0.039)	-0.060	0.093
Stress	-0.039 (0.022)	-0.081	0.004
Receiving emotional support	0.100 (0.020)***	0.062	0.139
Economic satisfaction	0.176 (0.049)***	0.080	0.272
Religiousness	0.031 (0.011)**	0.010	0.052
**Time**	0.002 (0.061)	-0.118	0.121
Sex (male)*time	-0.010 (0.008)	-0.026	0.006
Work (yes) *time	-0.012 (0.009)	-0.031	0.006
Ethnicity (Hakka) *time	-0.024 (0.009)*	-0.042	-0.006
Ethnicity (mainlander) *time	0.018 (0.014)	-0.009	0.045
Ethnicity (others) *time	0.001 (0.027)*	-0.053	0.054
Age (ordinal) *time	-0.025 (0.003)***	-0.030	-0.020
Education (ordinal) *time	0.011 (0.004)**	0.005	0.0184
Self-rated health	-0.006 (0.004)	-0.015	0.002
IADL disability number*time	-0.005 (0.004)	-0.013	0.002
Contact outside households*time	0.015 (0.004)***	0.007	0.022
Stress*time	0.004 (0.002)	-0.001	0.008
Receiving emotional support*time	-0.003 (0.001)	-0.006	0.001
Economic satisfaction*time	-0.009 (0.005)	-0.019	1.506E-5
Religiousness*time	0.0004 (0.001)	-0.002	0.002
**City-level indicators**			
Population density	0.009 (0.003)**	0.003	0.015
Median income	-0.009 (0.006)	-0.021	0.002
Unemployment	-0.056 (0.041)	-0.137	0.026
Population density*time	-0.0004 (0.0002)	-0.001	2.244E-5
Median income*time	0.001 (0.005)	1.167E-6	0.002
Unemployment*time	0.007 (0.009)	-0.011	0.024
**Random effect**			
Repeated	3.261 (0.056)		
Intercept (city)	1.717 (0.078)		
**Model fit**			
-2 log likelihood	42,501.360		
AIC	42,595.360		
BIC	42,609.737		

Note: Observations = 12,040. AIC: Akaike’s Information Criterion; BIC: Schwarz’s Bayesian Criterion. Reference groups: sex (female), work (no), ethnicity (Fuchien); other variables are ordinal or continuous. *p* < 0.1, *p < 0.05, **p < 0.001, ***p < 0.001

Table 2 shows the results of the linear mixed model of older adults’ cognitive function with individual-level and city-level indicators over time. The older participants had higher cognitive function at the intercept if they were working (β = 0.294), were not in a minority group (β =  − 1.361 for other groups), were younger (β =  − 0.205), were highly educated (β = 0.631), had better self-rated health (β = 0.174), had fewer AIDL physical difficulties (β =  − 0.231), received more emotional support (β = 0.100), had better economic satisfaction (β = 0.176), were more religious (β = 0.031), and lived in a city with a higher population density (β = 0.009). The factors causing cognitive function to decline faster on the time slope included being belonging to the Hakka ethnic group (β =  − 0.024) and being older (β =  − 0.025), while being highly educated (β = 0.011), having more social contacts outside the household (β = 0.015), and belonging in to other ethnic group (β = 0.001) may be protective of cognitive function over time.

Furthermore, we tried to use only the 8-item SPMSQ score as the cognitive function measure (please see Table S4). The 8 items were from a single scale (SPMSQ), but the score may be a less sensitive tool to measure mild cognitive impairment. The results were similar to Table 2, except that some of the variables (work, ethnicity, self-rated health, receiving emotional support, economic satisfaction, religiousness, contacts outside the household over time) became nonsignificant, and sex and median income over time became significant. The social support variables showed less predictive effect in more severe cognitive impairment.

## Discussion

This study used longitudinal nationally representative data to examine the effects of person, place, time, and their interactions on cognitive function in older adults in Taiwan. The individual’s protective factors for cognitive function at the intercept included being younger, working, not belonging to a minority group, being highly educated, having better self-rated health, receiving more emotional support, having better economic satisfaction, and being more religious. The protective environmental factor was living in a city with a higher population density. Being highly educated and having more social connectedness were protective over time.

### Individual factors

Higher education has been found to be a protective factor [4–6], and we found the same results in our study. We also found that the protection of higher education persisted over time. For the current older cohorts in Taiwan, the education level is relatively low compared with younger cohorts. However, the middle-aged and younger generations have better educational opportunities, and thus, the protective effect of education is expected. Work and financial satisfaction were also positively related to better cognitive function, consistent with previous research [2, 4–8]. The ethnicity difference in cognitive function also reflects the differences in socioeconomic status. Better socioeconomic status (including work, financial status, and education) affords better health-related opportunities in life, and thus, it has protective effects on cognitive function. Furthermore, continuity of work provides more chances in intellectual stimulation and more interaction with people. Thus better socioeconomic status would also be beneficial for cognitive function.

Many studies indicate that not only is receiving social support beneficial for mental health [26–28] but also that providing social support can also be protective for cognitive function [47]. We found that receiving more emotional social support was related to better cognitive function but providing or receiving instrumental support was not significant. Social support shows a protective effect on health outcomes through stress prevention, stress buffering, and direct effects [48]; thus, receiving emotional support would be related to better cognitive function. In addition, we also found that having more social contacts was also be protective of cognitive function over time. More social interaction with other people may stimulate intellectual performance and social relationships for older people, especially interactions with social contacts outside households. More social participation is encouraged to maintain cognitive function. However, providing instrumental support may be more related to obligation and burden for older people, and receiving instrumental support also represents worsening physical function. Therefore, providing and receiving instrumental support may not be related to positive cognitive function.

Being more religious was related to better cognitive function, consistent with previous longitudinal findings [25]. It is possible that religiousness would not only reduce stress but also encourage a healthy lifestyle and more participation in religious groups such that healthy behaviors and social participation are both beneficial for cognitive function.

Self-rated health and physical function were related to cognitive function as seen in previous studies [2, 7, 8, 18]. We used chronic diseases but not specific diseases in this study because that was not the aim of this study. However, chronic disease number was not significant. It is possible that not all the chronic diseases were related to cognitive function, and the added effect offset each other. Previous studies also suggest that health behaviors may affect cognitive function [5, 7, 21, 22]. We also included health behaviors (smoking and exercise) in the model, but they were not significant. It is possible that the effects of health behaviors were explained by the health conditions.

### City factors

Living in a city with a higher population density was found to be related to higher cognitive function at the intercept, even though the individual factors were included for control. This finding is consistent with previous studies [2, 31, 34]. An environment with a larger population represents an urban and developed environment. Cities with a higher population density may have well-developed infrastructure and barrier-free transportation environment for older people, and more social activities are available in cities. There are better opportunities for social participation, and therefore, the environment is beneficial for cognitive function. In addition, a higher percentage of highly educated people was found to be related to cognitive function. We did not include this variable because it was highly correlated with population density. Better educated older adults may move to larger cities to obtain better compensation and increase their work opportunities. However, the migration effect was also not significant when considering the city factors in the model. The direction of migration and highly educated population and their effects on cognitive function should be clarified through dynamic analysis of longitudinal data in future research.

### Limitations

There were several limitations in this study. First, the data were secondary data. The measures of cognitive function of older participants were not all consistent across waves. Only the consistent items of cognitive function across the waves of TLSA were used to define cognitive function. Second, not all the area-level factors were available in the early years, or not all the city-level variables in the data cover all the residences of the participants. For example, we planned to use secondary data that included social trust at the city level. However, the sampling of that data did not cover all cities in Taiwan. Thus, the data were not suitable to match the individual TLSA data. Third, the area-level indicators were measured at the city level, not a smaller unit such as a district or town. This is because most of the open data released by the government are based on cities/counties, not smaller units. However, the age-friendly policy in Taiwan is the city/county government’s responsibility. Consideration of area-level factors at the city level may show the effectiveness of the policy enacted by city governments. Fourth, some of the older adults migrated from one city to another during the follow-ups. The city-level effect may need a longer time to affect health outcomes, but the data were investigated every 3 or 4 years. We could not confirm the migration year. However, we added a migration time variable to adjust the migration effect.

## Conclusion

From an ecological viewpoint, a socially connected and secure environment at the individual level and at the city level may be related to cognitive function in older people. Having more social connectedness and support, being more religious, and living in cities with higher population densities are related to social interactions and participation, while higher education is related to socioeconomic status. Older adults are encouraged to maintain an active lifestyle and social connectedness with family, friends, neighborhoods or their community. A more social integrated lifestyle and financially secure living situation is suggested in the policy. Local government should create an age-friendly environment that can secure financial needs and encourage social interactions and participation to maintain cognitive function and promote active aging in older people.

## Supplementary Information

**Additional file 1: FigureS1**. Thesample of TLSA **Table S1**. Descriptive analysis of the city/county characteristicsacross 5 waves, 1999-2015 **TableS2.** Correlations of city-level indicators by year of 22 cites in Taiwan **Table S3.** Mixedlinear modeling of older adults’ cognitive function with individual and cityindicators by TLSA 1999-2015 (cognitive function measured by 10 items) **Table S4.** Multi-levelmixed linear modeling of older adults’ cognitive function with individual andcity indicators by TLSA 1999-2015 (cognitive function measured by 8 items ofSPMSQ)

## Data Availability

The data that support the findings of this study are available from by the Health and Welfare Data Science Center (HWDC), Ministry of Health and Welfare, R.O.C. (Taiwan) but restrictions apply to the availability of these data, which were used under license for the current study, and so are not publicly available. We do not have right to share the data. Data are however available from the authors upon reasonable request and with permission of the HWDC.
