# Supplementary material for: Individual and environmental factors associated with cognitive function in older people: a longitudinal multilevel analysis
Source: BMC Geriatr. 2022 Mar 23;22:243. doi: 10.1186/s12877-022-02940-9 (PMC8941778; doi:10.1186/s12877-022-02940-9)
Supplement: Supplementary file 1 — Additional file 1: FigureS1. Thesample of TLSA Table S1. Descriptive analysis of the city/county characteristicsacross 5 waves, 1999-2015 TableS2. Correlations of city-level indicators by year of 22 cites in Taiwan Table S3. Mixedlinear modeling of older adults’ cognitive function with individual and cityindicators by TLSA 1999-2015 (cognitive function measured by 10 items) Table S4. Multi-levelmixed linear modeling of older adults’ cognitive function with individual andcity indicators by TLSA 1999-2015 (cognitive function measured by 8 items ofSPMSQ) [file 12877_2022_2940_MOESM1_ESM.docx]

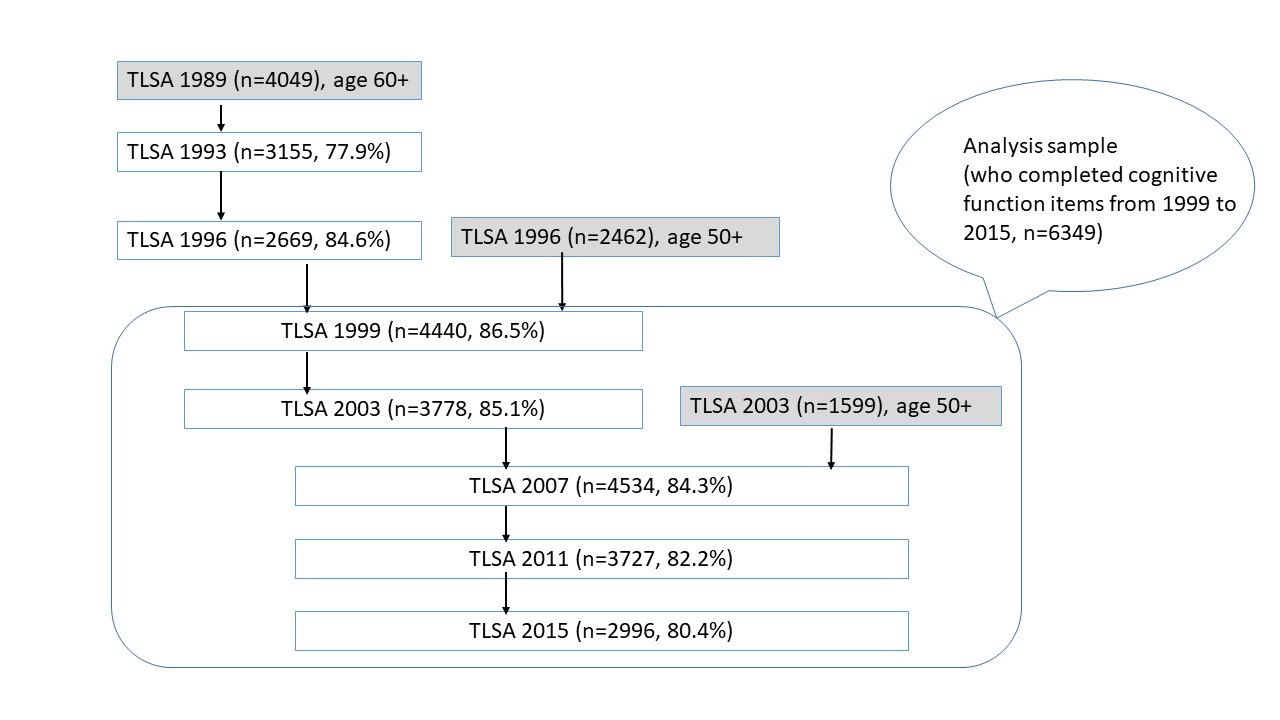


Figure S1. The sample of TLSA

Table S1. Descriptive analysis of the city/county characteristics across 5 waves, 1999-2015

| City-level indicators | 1999 | 2003 | 2007 | 2011 | 2015 |
| --- | --- | --- | --- | --- | --- |
| Population density (100 kilometer^2^) | 14.20 (21.88) | 14.53 (21.91) | 14.79 (22.00) | 15.02 (22.18) | 15.33 (22.52) |
| Older people % | 9.74% | 10.55% | 11.43% | 11.90% | 13.1% |
| High education % | 19.32% | 23.56% | 28.65% | 33.20% | 36.83% |
| Medical personnel (per 10 thousand population) | 65.14 (26.01) | 77.59 (26.96) | 86.79 (32.53) | 99.78 (37.69) | 109.85 (41.44) |
| Hospital beds (per 10 thousand population) | 58.77 (24.89) | 65.40 (24.38) | 68.52 (26.34) | 71.35 (27.77) | 69.99 (26.55) |
| Green land | 5.77 (6.42) | 4.12 (3.82) | 5.28 (5.48) | 5.80 (5.12) | 7.03 (4.98) |
| Crime (per 100 thousands population) | 833.74 (272.98) | 704.47 (189.60) | 1039.39 (321.70) | 1045.99 (276.46) | 1164.04 (329.43) |
| Low income rate | 0.91% | 1.21% | 1.30% | 1.55% | 1.53% |
| Median income (10 thousands NT) | 72.45 (15.05) | 70.53 (15.73) | 72.79 (16.84) | 72.55 (17.20) | 76.36 (15.18) |
| Unemployment% | 2.53% | 4.72% | 3.80% | 4.09% | 3.53% |
| Gini | 0.3100 (0.0277) | 0.3203 (0.0247) | 0.3286 (0.0316) | 0.3300 (0.0240) | 0.3348 (0.0258) |

Table S2. Correlations of city-level indicators by year of 22 cites in Taiwan

| 1999 | population density | elderly percentage | medical professional | bed no. | crime rate | low income rate | median income NT | unemployment rate | High education | Gini |
| --- | --- | --- | --- | --- | --- | --- | --- | --- | --- | --- |
| population density | 1 |  |  |  |  |  |  |  |  |  |
| elderly percentage | -.288 | 1 |  |  |  |  |  |  |  |  |
| medical professional | .654^**^ | -.343 | 1 |  |  |  |  |  |  |  |
| bed no. | .307 | -.143 | **.790^**^** | 1 |  |  |  |  |  |  |
| crime rate | .314 | -.049 | .221 | .407 | 1 |  |  |  |  |  |
| low income rate | -.244 | **.703^**^** | -.206 | .042 | .136 | 1 |  |  |  |  |
| median income NT | .529^*^ | -.466^*^ | .371 | -.031 | -.296 | -.668^**^ | 1 |  |  |  |
| unemployment rate | .204 | -.525^*^ | .274 | .377 | .515^*^ | -.306 | -.121 | 1 |  |  |
| high education | **.855^**^** | -.442^*^ | **.729^**^** | .322 | .074 | -.426^*^ | **.717^**^** | .119 | 1 |  |
| Gini | -.168 | .459^*^ | .137 | .407 | .262 | .650^**^ | -.690^**^ | .144 | -.252 | 1 |
| 2003 | population density | elderly percentage | medical professional | bed no. | crime rate | low income rate | median income NT | unemployment rate | high education | Gini |
| population density | 1 |  |  |  |  |  |  |  |  |  |
| elderly percentage | -.265 | 1 |  |  |  |  |  |  |  |  |
| medical professional | .692^**^ | -.318 | 1 |  |  |  |  |  |  |  |
| bed no. | .324 | -.118 | **.838^**^** | 1 |  |  |  |  |  |  |
| crime rate | .358 | .208 | .506^*^ | .525^*^ | 1 |  |  |  |  |  |
| low income rate | -.177 | .547^**^ | -.032 | .112 | .101 | 1 |  |  |  |  |
| median income NT | .566^**^ | -.500^*^ | .392 | .120 | -.297 | -.557^**^ | 1 |  |  |  |
| unemployment rate | .106 | -.166 | .096 | .064 | .419 | .057 | -.456^*^ | 1 |  |  |
| high education | **.875^**^** | -.516^*^ | **.760^**^** | .394 | .281 | -.347 | .666^**^ | .079 | 1 | -.446^*^ |
| Gini | -.341 | .498^*^ | -.019 | .193 | .057 | .671^**^ | -.575^**^ | .027 | -.446^*^ | 1 |
| 2007 | population density | elderly percentage | medical professional | bed no. | crime rate | low income rate | median income NT | unemployment rate | high education | Gini |
| population density | 1 |  |  |  |  |  |  |  |  |  |
| elderly percentage | -.226 | 1 |  |  |  |  |  |  |  |  |
| medical professional | .653^**^ | -.198 | 1 |  |  |  |  |  |  |  |
| bed no. | .305 | -.042 | **.870^**^** | 1 |  |  |  |  |  |  |
| crime rate | .311 | -.148 | .694^**^ | .675^**^ | 1 |  |  |  |  |  |
| low income rate | -.157 | .473^*^ | .063 | .167 | -.045 | 1 |  |  |  |  |
| median income NT | .580^**^ | -.635^**^ | .140 | -.150 | -.170 | -.540^**^ | 1 |  |  |  |
| unemployment rate | .080 | .066 | .381 | .303 | **.770^**^** | .135 | -.370 | 1 |  |  |
| high education | **.898^**^** | -.468^*^ | **.709^**^** | .375 | .438^*^ | -.316 | .646^**^ | .149 | 1 |  |
| Gini | -.231 | .609^**^ | -.018 | .196 | -.080 | .592^**^ | -.596^**^ | -.176 | -.351 | 1 |
| 2011 | population density | elderly percentage | medical professional | bed no. | crime rate | low income rate | median income NT | unemployment rate | high education | Gini |
| population density | 1 |  |  |  |  |  |  |  |  |  |
| elderly percentage | -.192 | 1 |  |  |  |  |  |  |  |  |
| medical professional | .651^**^ | -.120 | 1 |  |  |  |  |  |  |  |
| bed no. | .313 | .042 | **.874^**^** | 1 |  |  |  |  |  |  |
| crime rate | .338 | .011 | .529^*^ | .496^*^ | 1 |  |  |  |  |  |
| low income rate | -.139 | .343 | -.006 | .085 | -.085 | 1 |  |  |  |  |
| median income NT | .624^**^ | -.642^**^ | .137 | -.166 | -.010 | -.444^*^ | 1 |  |  |  |
| unemployment rate | .142 | .218 | .403 | .314 | .607^**^ | .211 | -.294 | 1 |  |  |
| high education | **.903^**^** | -.422 | **.730^*^**^*^ | .389 | .449^*^ | -.276 | .693^**^ | .196 | 1 |  |
| Gini | -.491^*^ | .689^**^ | -.132 | .181 | -.054 | .354 | -.627^**^ | -.053 | -.546^*^ | 1 |
| 2015 | population density | elderly percentage | medical professional | bed no. | crime rate | low income rate | median income NT | unemployment rate | High education | Gini |
| population density | 1 |  |  |  |  |  |  |  |  |  |
| elderly percentage | -.084 | 1 |  |  |  |  |  |  |  |  |
| medical professional | .632^**^ | .060 | 1 |  |  |  |  |  |  |  |
| bed no. | .345 | .193 | **.887^**^** | 1 |  |  |  |  |  |  |
| crime rate | .219 | .560^**^ | .503^*^ | .640^**^ | 1 |  |  |  |  |  |
| low income rate | -.112 | .299 | .095 | .161 | .368 | 1 |  |  |  |  |
| median income NT | .677^**^ | -.568^**^ | .259 | -.016 | -.235 | -.522^*^ | 1 |  |  |  |
| unemployment rate | .124 | .395 | .428^*^ | .386 | .612^**^ | .199 | -.130 | 1 |  |  |
| high education | **.918^**^** | -.258 | **.723^**^** | .442^*^ | .148 | -.184 | **.754^**^** | .188 | 1 |  |
| Gini | -.395 | .362 | -.141 | .097 | .425 | .518^*^ | -.632^**^ | -.159 | -.463^*^ | 1 |

Table S3. Mixed linear modeling of older adults’ cognitive function with individual and city indicators by TLSA 1999-2015 (cognitive function measured by 10 items)

| Variables | Model S1. Individual factors at intercept and time slope  B (SE) | Model S2: City indicators at intercept and time slope  B (SE) |
| --- | --- | --- |
| **Fixed effects** |  |  |
| **Individual-level** |  |  |
| Intercept | 9.850 (0.511)*** | 15.929 (2.214)*** |
| Sex (male) | 0.182 (0.119) |  |
| Marital status (having spouse) | 0.156 (0.113) |  |
| Children (yes) | -0.090 (0.383) |  |
| Living arrangement (alone) | -0.061 (0.185) |  |
| Smoking (no) | -0.023 (0.126) |  |
| Smoking (quitted) | -0.063 (0.145) |  |
| Drinking alcohol | -0.051 (0.029) |  |
| Exercise (regular) | 0.009 (0.084) |  |
| Work (yes) | 0.193 (0.096)* |  |
| Social group participation (yes) | 0.125 (0.082) |  |
| Ethnicity (Hakka) | -0.012 (0.108) |  |
| Ethnicity (mainlander) | 0.232 (0.146) |  |
| Ethnicity (others) | -0.977 (0.319)** |  |
| Age (ordinal) | -0.274 (0.030)*** |  |
| Education (ordinal) | 0.542 (0.040)*** |  |
| Disease number | 0.004 90.037) |  |
| Self-rated health | 0.161 (0.047)** |  |
| ADL disability no. | 0.025 (0112) |  |
| IADL disability no. | -0.311 (0.066)*** |  |
| Nagi function difficulties | -0.027 (0.015) |  |
| Contact outside households | 0.104 (0.037)** |  |
| Depressive symptoms | -0.010 (0.010) |  |
| Stress | -0.058 (0.023)* |  |
| Providing instrumental help | -0.042 (0.041) |  |
| Receiving emotional support | 0.097 (0.024)*** |  |
| Receiving instrumental support | -0.094 (0.054) |  |
| Economic satisfaction | 0.135 (0.049)** |  |
| Religiousness | 0.038 (0.010)*** |  |
| Migration times | 0.194 (0.098)* |  |
| **Time** | 0.108 (0.058) | -0.176 (0.181) |
| **Time square** | -0.001 (0.001) | -0.016 (0.002)*** |
| Sex (male)*time | -0.030 (0.012)** |  |
| Marital status (having spouse) *time | -0.022 (0.012) |  |
| Children (yes) *time | 0.001 (0.045) |  |
| Living arrangement (alone) *time | 0.017 (0.017) |  |
| Smoking (no) *time | -0.004 (0.013) |  |
| Smoking (quitted) *time | 0.019 (0.015) |  |
| Drinking alcohol*time | 0.005 (0.003) |  |
| Exercise regular*time | 0.006 (0.008) |  |
| Work (yes) *time | -0.006 (0.010) |  |
| Social group participation (yes) *time | 0.003 (0.008) |  |
| Ethnicity (Hakka) *time | -0.013 (0.010) |  |
| Ethnicity (mainlander) *time | 0.002 (0.015) |  |
| Ethnicity (others) *time | -0.006 (0.031) |  |
| Age (ordinal) *time | -0.014 (0.003)*** |  |
| Education (ordinal) *time | 0.013 (0.004)** |  |
| Disease number*time | -0.001 (0.003) |  |
| Self-rated health | -0.015 (0.005)** |  |
| ADL disability number*time | 0.010 (9.009) |  |
| IADL disability number*time | 0.002 (0.005) |  |
| Nagi function difficulties*time | -0.003 (0.001)* |  |
| Contact outside households*time | 0.006 (0.004) |  |
| Depressive symptoms*time | -0.001 (0.001) |  |
| Stress*time | 0.007 (0.002)** |  |
| Providing instrumental help*time | -0.002 (0.002) |  |
| Receiving emotional support*time | 0.004 (0.006) |  |
| Receiving instrumental support*time | -0.006 (0.005) |  |
| Economic satisfaction*time | -0.008 (0.001) |  |
| Religiousness*time | -0.023 (0.001) |  |
| Migration*time | -0.023 (0.009)* |  |
| **City-level indicators** |  |  |
| Population density |  | 0.029 (0.008)*** |
| Older people percentage |  | -0.132 (0.077) |
| Hospital beds |  | 0.002 (0.006) |
| Crime rate |  | -0.0004 (0.0004) |
| Low income rate |  | -0.345 (0.179) |
| Median income |  | -0.044 (0.014)** |
| Unemployment |  | -0.241 (0.080)** |
| Greenland |  | -0.012 (0.022) |
| Gini |  | -6.666 (4.765) |
| Population density*time |  | -0.001 (0.001)* |
| Older people *time |  | 0.009 (0.005) |
| Hospital beds*time |  | -0.0004 (0.0004) |
| Crime rate*time |  | <0.001 (<0.001) |
| Low income rate*time |  | 0.014 (0.013) |
| Median income*time |  | 0.003 (0.001)** |
| Unemployment*time |  | 0.025 (0.014) |
| Greenland*time |  | -0.003 (0.002) |
| Gini*time |  | 0.060 (0.468) |
| **Random effect** |  |  |
| Repeated | 4.856 (0.065) | 7.529 (0.105) |
| Intercept (city) | 0.088 (0.034) | 0.211 (0.097) |
| **Model fit** |  |  |
| -2 log likelihood | 50783.591 | 50655.473 |
| AIC | 50787.591 | 50659.473 |
| BIC | 50802.263 | 50673.966 |

Note: Observations=12040. AIC: Akaike’s Information Criterion; BIC: Schwarz’s Bayesian Criterion. Reference groups: sex (female), marital stats (no spouse), children (no), living arrangement (with others), exercise (irregular), work (no), social group participation (no), ethnicity (Fuchien); other variables are ordinal or continuous. p<0.1, *p<0.05, **p<0.001, ***p<0.001.

Table S4. Multi-level mixed linear modeling of older adults’ cognitive function with individual and city indicators by TLSA 1999-2015 (cognitive function measured by 8 items of SPMSQ)

|  |  | 95% C.I. | |
| --- | --- | --- | --- |
| Variables | B (SE) | Lower | Upper |
| **Fixed effects** |  |  |  |
| **Individual-level** |  |  |  |
| Intercept | 7.459 (0.237)*** | 6.995 | 7.923 |
| Sex (male) | 0.126 (0.043)** | 0.042 | 0.211 |
| Work (yes) | 0.067 (0.040) | -0.012 | 0.146 |
| Ethnicity (Hakka) | 0.040 (0.049) | -0.057 | 0.137 |
| Ethnicity (mainlander) | 0.102 (0.070) | -0.035 | 0.238 |
| Ethnicity (others) | -0.262 (0.138) | -0.532 | 0.009 |
| Age (ordinal) | -0.009 (0.013) | -0.034 | 0.017 |
| Education (ordinal) | 0.073 (0.019)*** | 0.036 | 0.109 |
| Self-rated health | 0.010 (0.018) | -0.025 | 0.046 |
| IADL disability | -0.187 (0.021)*** | -0.229 | -0.145 |
| Contact outside households | 0.018 (0.016) | -0.0139 | 0.049 |
| Stress | -0.014 (0.009) | -0.032 | 0.003 |
| Receiving emotional support | 0.015 (0.008) | -0.001 | 0.031 |
| Economic satisfaction | -0.018 (0.020) | -0.057 | 0.022 |
| Religiousness | 0.004 (0.004) | -0.005 | 0.013 |
| **Time** | -0.043 (0.025) | -0.092 | 0.006 |
| Sex (male)*time | 0.012 (0.003)** | 0.005 | 0.018 |
| Work (yes) *time | -0.007 (0.004) | -0.014 | 0.001 |
| Ethnicity (Hakka) *time | -0.003 (0.004) | -0.010 | 0.005 |
| Ethnicity (mainlander) *time | -0.001 (0.006) | -0.0117 | 0.011 |
| Ethnicity (others) *time | -0.020 (0.011) | -0.042 | 0.002 |
| Age*time | -0.011 (0.001)*** | -0.013 | -0.009 |
| Education (ordinal) *time | 0.011 (0.001)*** | 0.009 | 0.014 |
| Self-rated health | -0.002 (0.002) | -0.005 | 0.002 |
| IADL disability number*time | -0.002 (0.002) | -0.005 | 0.001 |
| Contact outside households*time | 0.003 (0.002) | -0.0003 | 0.006 |
| Stress*time | 0.001 (0.001) | -0.0005 | 0.003 |
| Receiving emotional support*time | 0.001 (0.001) | -0.0009 | 0.002 |
| Economic satisfaction*time | 0.004 (0.002)* | 7.414E-5 | 0.008 |
| Religiousness*time | 0.007 (0.001) | -0.0001 | 0.002 |
| **City-level indicators** |  |  |  |
| Population density | 0.003 (0.001)** | 0.001 | 0.006 |
| Median income | -0.005 (0.003) | -0.010 | 0.0002 |
| Unemployment | 0.027 (0.017) | -0.007 | 0.060 |
| Population density*time | -0.0001 (9.854E-5) | -0.0004 | 7.497E-6 |
| Median income*time | 0.0004 (0.0002)* | 4.105E-5 | 0.001 |
| Unemployment*time | -0.005 (0.004) | -0.012 | 0.002 |
| **Random effect** |  |  |  |
| Repeated | 0.553 (0.010) |  |  |
| Intercept (city) | 0.305 (0.014) |  |  |
| **Model fit** |  |  |  |
| -2 log likelihood | 25309.509 |  |  |
| AIC | 25313.509 |  |  |
| BIC | 25327.886 |  |  |

Note: Observations=12040. AIC: Akaike’s Information Criterion; BIC: Schwarz’s Bayesian Criterion. Reference groups: sex (female), work (no), ethnicity (Fuchien); other variables are ordinal or continuous. p<0.1, *p<0.05, **p<0.001, ***p<0.001.
